# Revised generic placement of *Brachypelma embrithes* (Chamberlin & Ivie, 1936) and *Brachypelma angustum* Valerio, 1980, with definition of the taxonomic features for identification of female *Sericopelma* Ausserer, 1875 (Araneae, Theraphosidae)

**DOI:** 10.3897/zookeys.526.6315

**Published:** 2015-10-12

**Authors:** Ray Gabriel, Stuart J. Longhorn

**Affiliations:** 1 Hope Entomological Collections, Oxford University Museum of Natural History (OUMNH), Parks Road, Oxford, England, OX1 3PW, United Kingdom

**Keywords:** Spider taxonomy, Theraphosidae, *Brachypelma*, transfer, *Sericopelma*

## Abstract

The tarantula genus *Sericopelma* was originally defined based on male specimens, most notably lacking tibial spurs on leg I. Early female specimens were unrecognised as *Sericopelma*, and typically placed in *Eurypelma* – a dumping ground for problem specimens. The first females were only later recognised, but authors failed to adequately define female *Sericopelma*. Here, the holotypes of the southern-most alleged *Brachypelma* species, *Brachypelma embrithes* (Chamberlin & Ivie, 1936) and *Brachypelma angustum* Valerio, 1980 were examined, and finding both to possess defining characteristics of *Sericopelma* were transferred. The taxonomic attributes to define *Sericopelma* relative to *Brachypelma* and select other Neotropical genera are discussed, especially for females. As important diagnostic characters for *Sericopelma*, the single (unilobar) spermathecae swollen at the apex forming a P-shaped cross-section, metatarsus IV with trace scopula, femur IV with a dense retrolateral pad of plumose hair, plus other attributes. Some past confusion in these characters are clarified and *Sericopelma* relative to *Brachypelma* and *Megaphobema mesomelas* are discussed. Finally recommendations are given about these taxonomic changes for CITES regulations.

## Introduction

*Sericopelma* Ausserer, 1875 was established for a male tarantula from an unspecified location in Panama without leg I tibial apophyses, named *Sericopelma rubronitens* Ausserer, 1875. Sericopelma was originally a subgenus of *Eurypelma* Koch, 1851, but later given full generic status (Simon 1892). Karsch (1880) also described an early male tarantula from Chiriquí Panama without leg I tibial apophyses as *Theraphosa panamana* Karsch, 1880. In revision, Simon (1892) synonymized *Theraphosa panamana* into Ausserer’s *Sericopelma rubronitens*, emphasizing the lack of male tibial apophyses. He also considered that another male in the Paris collection from Chiriquí, Panama, might be the same. Like Karsch, he drew on similarities to the genus *Theraphosa*, where males of *Theraphosa blondi* (Latreille, 1804) also lack tibial apophyses, but distinguished the genera by several other features such as bulb shape, eye ratios, and cephalothorax dimensions. Soon after, Pickard-Cambridge (1897) described another species from four males also collected around Chiriquí province in Panama, which he named *Sericopelma commune* F.O.P.-Cambridge, 1897. He distinguished *Sericopelma* by femur IV “*with a thick scopuliform pad on inner side*”, male tibia I without spurs, and emphasized the lack of scopulae on protarsus (metatarsus) of leg IV “*with no thick scopulae on the inner side*”. Pocock (1901) again treated *Sericopelma* as congeneric with *Theraphosa*, but was not subsequently accepted.

Throughout the early twentieth century, only male *Sericopelma* were formally known and females remained unrecognised. Simon had described *Eurypelma panamense* Simon (1891) from a female with the vague locality of “Panama, Guatemala” emphasising conspicuous scopulae on femural leg IV, but failed to recognize it as *Sericopelma* (see Gabriel 2009). Schiapelli and Gerschman de Pikelin (1967) then evaluated both sexes of a *Sericopelma* sp. from “Rio Grande, Nicaragua” (? = Río Grande de Matagalpa) and illustrated the first female spermathecae. Next, Valerio (1980) described seven new Costa Rican species including three from both sexes, namely *Sericopelma generala*, *Sericopelma immensum* and *Sericopelma silvicola*, but only males for *Sericopelma dota*, *Sericopelma ferrugineum*, *Sericopelma melanotarsum* and *Sericopelma upala*. Following Schiapelli and Gerschman (1967), *Sericopelma* was characterized in Valerio (1980) by the “*presence of a thick scopula on the inner side of femur IV, and by the absence of spurs on tibia I [of males], and by the absence of stridulatory setae on trochanter I, and [absence of] scopula on metatarsus IV*”. Smith (1991b) then re-described a syntype male of *Sericopelma commune* and illustrated the spermathecae of a female *Sericopelma* sp. in the BMNH collection. He suggested the latter was the un-described female of *Sericopelma commune*, and although stating “not a species description”, it has been subsequently treated as such (*i.e.* World Spider Catalog 2015). We deduce that Smith (1991b) was referring to a female from Pozo Azul de Pirrís, Costa Rica, assigned by Valerio to *Sericopelma immensum* [see discussion]. Schmidt (1994) described the exuvia of a female as *Sericopelma melanotarsum*, illustrating the spermathecae, but did not give collection locality nor list any museum deposit. Most recently, Gabriel (2009) transferred the Panamanian *Sericopelma panamense* (Simon, 1891) from *Eurypelma*, illustrating the holotype spermathecae plus of another Panamanian *Sericopelma* sp. from Boquete, Chiriquí province, whilst Gabriel and Longhorn (2011) illustrated the spermathecae of a *Sericopelma* sp. from Bocas del Toro province, Panama. Finally Andre and Esche (2011) showed the spermathecae of *Sericopelma melanotarsum* alongside other morphological data, plus substantial ecological, behavioural, and captive breeding data. However, despite these studies, *Sericopelma* as a whole remains poorly defined.

The genus *Brachypelma* Simon, 1891 was created for *Mygale emilia* White, 1856, originally listed from Panama. However, this location is erroneous, as the natural distribution of the type species and allies is South-western México (*i.e.* Smith 1994, Locht et al. 1999, Schmidt 2003). *Brachypelma* is currently said to range from México to Panama, though the southern-most species have not been revised until now. Pickard-Cambridge (1897) could not distinguish *Brachypelma* from *Eurypelma*, and considered the genera synonymous, describing other species such as *Brachypelma smithi* (F.O.P.-Cambridge, 1897), which has become a flagship for conservation efforts under the Convention on International Trade in Endangered Species (CITES). *Eurypelma* was partly dismembered by Pocock (1903), who recognised the importance of plumose hairs on leg I and palp to define *Brachypelma*, whilst Simon (1903) admitted *Eurypelma* was previously insufficiently characterised. *Brachypelma* was considered valid by Valerio (1980) who described three new species from Costa Rica, *Brachypelma albopilosum* Valerio, 1980, *Brachypelma fossorium* Valerio, 1980 and *Brachypelma angustum* Valerio, 1980. Soon after, Smith (1986) formalised additional transfers from *Eurypelma* to *Brachypelma*. Valerio (1980) had previously also transferred the Costa Rican *Eurypelma mesomelas* O.P.-Cambridge, 1892 into *Brachypelma* and described the female. Smith (1986, 1987) agreed, but not Schmidt (1991a/b), who further transferred it to *Megaphobema* despite objections by Smith (1991a/b). Schmidt (1993, 2003) continued to list this as *Megaphobema mesomelas*, as does the current World Spider Catalog (World Spider Catalog 2015).

Here taxonomic placement of some Costa Rican and Panamanian species is re-evaluated. Petrunkevitch (1925) had previously recorded several alleged *Eurypelma* from Panama, listing some as species now placed in *Brachypelma* (namely *emilia*, *sabulosum* and *vagans*) since known only from México, Guatemala and Belize (Smith 1994, Locht et al. 1999). Chamberlin and Ivie (1936) went further and described a species from Barro Colorado Island [Panama] as *Eurypelma embrithes*, placing it in that genus without explanation. Already, the robustness of *Eurypelma* should have been suspicious, as many species had been placed there without justification. Petrunkevitch (1939) later considered *Eurypelma* as “*genus incertum* and *invalidum*”, although was treated as valid by Roewer (1942). Raven (1985) went on to regard *Eurypelma* as a junior synonym of the arboreal *Avicularia* Lamarck, 1818. Consequently several species were transferred to *Avicularia* that clearly did not belong there. Schmidt (1993) instead transferred several former *Eurypelma* into *Aphonopelma*, leading to the new combination *Aphonopelma embrithes* (Chamberlin & Ivie, 1936) although gave no justification, nor apparently examined any relevant types. Smith (1994) relocated *embrithes* to *Brachypelma* after reviewing many historical specimens, but did not explain his placement of this Panamanian species, thereby becoming the southern-most representative of *Brachypelma*. However, *Brachypelma embrithes* has since been listed as such (*e.g.* World Spider Catalog 2015) and receives legal protection under CITES legislation. However, much taxonomic revision is necessary for this protected genus in the context of others genera such as *Sericopelma*, although Smith (1994), Locht et al. (1999) and West (2005) have each made valuable contributions. Here, type material of *Brachypelma embrithes* and *Brachypelma angustum* are re-examined and their taxonomic placement is reconsidered in a modern context.

## Methods

Specimens were examined under a binocular microscope, photographs of spermathecae and other structures were typically made using a Leica M135 auto-montage system, other photographs with a Fujipix S5000. All measurements are given in millimetres (mm). *Abbreviations, Institutes*: AMNH = American Museum of Natural History; BMNH = British Museum of Natural History; CNAN = Colección Nacional de Arácnidos, Instituto de Biología, Universidad Nacional Autónoma de México; LAAHFC = Laboratorio de Acarología “Anita Hoffmann”, Facultad de Ciencias, Universidad Nacional Autónoma de México; MCZ = Museum of Comparative Zoology Harvard; MIUCR = Museo de Invertebrados University Costa Rica, MIUP = Museo de Invertebrados G.B. Fairchild, Universidad de Panama; MNHN = Muséum National d’Histoire Naturelle, Paris; OUMNH = Oxford University Museum of Natural History, UK; PMY = Peabody Museum of Natural History, Yale, Connecticut; SJLC = Private collection Stuart J. Longhorn; STRI = Smithsonian Tropical Research Institute; NHMV = Natural History Museum Vienna (Naturhistorisches Museum Wien), Austria; ZMB = Museum für Naturkunde, Berlin, Germany. *Others*: CITES = Convention on International Trade in Endangered Species; ANAM = Autoridad Nacional del Ambiente; B.C.I. = Barro Colorado Island; Imm = immature specimen; Ident. = indeterminate; det. = determined as; ALE = Anterior Lateral Eyes; PLE = Posterior Lateral Eyes; AME = Anterior Medial Eyes, PLE = Posterior Lateral Eyes; LHS = Left Hand side (from above); RHS = Right Hand Side. DMS = Degrees, Minutes, Seconds. Authors comments/emphases in[ ].

**Type material examined**: 1 ♀ holotype & 1 imm ♀ paratype *Aphonopelma seemanni* F.O.P-Cambridge 1897, BMNH [unknown accession], Puerto Culebra, Costa Rica, leg. Dr. B. Seemann; 1 ♀ holotype *Brachypelma angustum* Valerio 1980, UCR-433; 1 ♂ holotype *Brachypelma baumgarteni* Smith 1993, BMNH 1999-122, Sierra Madre del Sur, Mexico, leg. M. Baumgarten; 1 ♀ holotype *Brachypelma embrithes* (Chamberlin and Ivie 1936), AMNH [No accession], Barro Colorado Island (B.C.I), Panama, leg. unknown; 1 ♂ neotype *Brachypelma emilia* (White 1856), BMNH 98-12-24-32, Ciudad (Durango, Mexico) leg. Mr. Forrer (See Smith 1994); 1 ♂ paraneotype *Brachypelma emilia* (labeled as paratype), OUNMH Jar 106, Ciudad, Mex (Durango, Mexico) leg. Forrer; 1 ♂ holotype *Brachypelma fossorium* Valerio 1980, UCR-238 Guanacaste, Gte Filadelfia, leg. 24 jul.1973, Eddie Herrera & 1 ♀ allotype UCR-126, Guanacaste, Finca Santo Tomás, leg. 9 Apr. 1966, C.E. Valerio; 1 ♀ holotype *Brachypelma sabulosum* (F.O.P.-Cambridge 1897) BMNH 1898.12.24.54, Tikal Petten (=Peten), Guatemala, leg. A.P.Maudslay; 1 j♂ holotype (originally listed as ♀) *Brachypelma smithi* (F.O.P.-Cambridge 1897), BMNH 1898.12.24.33 (1143), Dos Arroyos, Mexico (=Guerrero), leg. H.H. Smith; 1 ♂ holotype & 1 ♀ paratype *Brachypelma vagans* (Ausserer 1875), BMNH 1890-7-1-380-282, Yucatan (Keyserling collection), leg. Unknown; 1 ♂ holotype *Megaphobema mesomelas* (O.P.-Cambridge 1892), BMNH 1898.12.24.55, Caché, Costa Rica, leg. H. Rogers, Goodman and Salvin collection [ex-dried]; 1 ♂ holotype, 1 ♂ paratype (=syntype) *Megaphobema robustum* (Ausserer 1875), BMNH 1890.7.1.369-371, Bogotá [=Colombia] (Keyserling collection), leg. unknown; 1 ♂ holotype *Megaphobema peterklaasi* Schmidt 1994, SMF 38028 Costa Rica, leg. P. Klaas, det G. Schmidt 1994 & 1 ♂ paratype SMF 38030, same data; 1 ♂ holotype & 1 ♀ allotype *Megaphobema velvetosoma* Schmidt 1995, SMF 57910, Ecuador, area around Tena, leg. D.Antonelli; 3 ♂ ‘syntypes’ (lectotype and paralectotypes) *Sericopelma commune* F.O.P.-Cambridge 1897, BMNH 1898.12.24 19-21, Panama, Chiriquí, leg. G.C. Champion; 1 ♂ paralectotype (fourth syntype) *Sericopelma commune* OUMNH Jar 106, Chiriquí, leg G. Champion.; 1 ♂ holotype *Sericopelma immensum* Valerio 1980, UCR-237, San José, Cantón Dota, Finca El Cedral 2100 m, leg. 28 Oct. 1972, Guillermo Solís & 1 ♀ allotype UCR-288, San José, Cantón Puriscal, Naranjal de Guarumal, 480 m, leg. 5 Apr. 1972, Luis E. Jirón; 1 ♀ holotype *Sericopelma panamense* (Simon 1891), AR 4850 MNHN (Simon Collection), ‘Panama and Guatemala’ leg. unknown; 1 ♂ holotype *Sericopelma panamana/um* (Karsch 1880), ZMB 2394 BERLIN = Junior synonym of *Sericopelma rubronitens* by Simon (1892), Panama, Chiriquí, leg. Unknown; 1 ♂ holotype *Sericopelma rubronitens* Ausserer 1875, NHMV Nr.1874.III.1, WIEN, Panama, leg. unknown.

**Other material examined**: See supplement for full listing of examined Nicaraguan, Costa Rican and Panamanian *Sericopelma* spp. in the collections at BMNH, MCZ, MNHN, MIUP, OUMNH, PMY, SJLC. Specimens of various *Brachypelma* sp. from BMNH, CNAN, MCZ, OUMNH, LAAHCF, SJLC, *Megaphobema* sp. from MCZ, OUMNH, SJLC and *Theraphosa* sp. from OUMNH and SJLC.

## Results

### Taxonomy Family Theraphosidae Thorell, 1869 Genus *Sericopelma* Ausserer, 1875

#### Sericopelma embrithes

Taxon classificationAnimaliaAraneaeTheraphosidae

(Chamberlin & Ivie, 1936) comb. n.

Eurypelma embrithes Chamberlin & Ivie, 1936: 7 (D female)
Avicularia embrithes Raven, 1985: 146, 148, 151 (T f from *Eurypelma*).
Aphonopelma embrithes Schmidt, 1993: 78 (T f from *Eurypelma* = *Avicularia*).
Brachypelma embrithes Smith, 1994: 160 (T f from *Eurypelma* = *Avicularia*).

##### Description.

**Figure 1–5.**
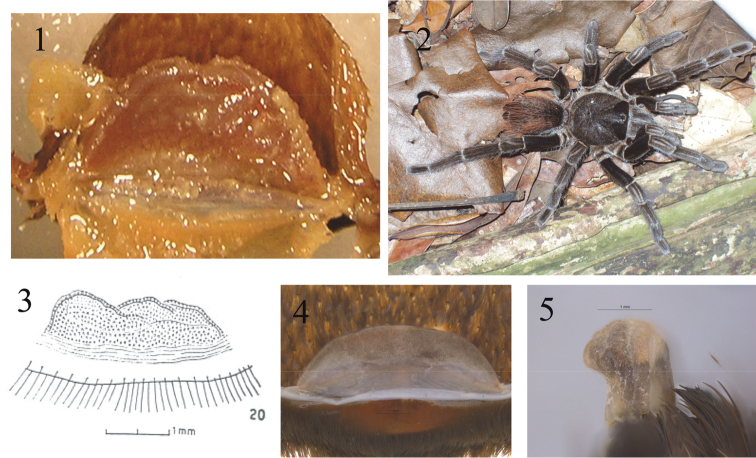
**1** Spermathecae from holotype of *Sericopelma embrithes* in dorsal view **2** Live specimen *in situ* of Sericopelma cf. embrithes at type locality on Barro Colorado Island, probable adult Female [Photo: Insa Wagner, STRI] **3** Spermatheca drawing of female Nicaraguan *Sericopelma* sp. in dorsal from Schiapelli and Gerschman (1967), their figure 20 **4** Spermatheca of mature female *Sericopelma* sp. Boquete (8.78°N, 82.43°W), Districto Boquete, Chiriquí Province, Panama, dorsal view lacking any distinct median notch **5** Same spermatheca (as 4) in lateral view with diagnostic ‘P-shape’ (of seen in reverse).

*Female* (Holotype AMNH): Total length including chelicerae 58.6. Carapace, length 27.6, width 23.2. Caput, high. Ocular tubercle, length 2.6, width 3. Anterior row procurved, posterior row recurved. Eyes ALE > AME, AME > PLE, PLE > PME. Clypeus; 0.9, clypeal fringe long. Fovea, deep transverse. Maxillae, with 100–120 cuspules, covering approximately 60% of proximal edge. Labium, length 3.2, width 4.4, with 40–60 labial cuspules most separated by less than 0.5 - 1 times the width of a single cuspule. Labio-sternal mounds separate. Sternum, damaged with three pairs of sigilla. Femur IV with a dense pad of plumose hair on retro-lateral surface, pro-lateral surfaces of trochanter/femur of anterior legs lacking stridulatory setae. Tarsi I–IV densely scopulate. Metatarsal scopulae, I 88%, II 83%, III 64%, IV 15% of the length of the segment, IV divided. Lengths of leg and palpal segments see Table 2. Spination: femurs I, III, IV, 0-0-2 palp d 0-0-1, patella I, palp, II, III 0-2-0, IV 0-3-0, tibia 1 d 0-2-0, v 0-0-3, II d 1-2-0, v 1-1-3, III d 2-2-2, v 0-2-3 (apical), tibia IV d 4-3-2, v 2-1-2, palp d 0-1-2, metatarsus I 0-0-1, II v 0-0-2 (apical), III d 2-3-2, v 4-0-5 (apical), IV d 3-2-2, v 5-5-9 (5 apical). Posterior lateral spinnerets, with three segments, basal 4.4, medial 3.7, digitiform apical 6.1.Lateral median spinnerets, with one segment. Spermathecae, single domed receptacle apically swollen (Fig. 1). Urticating hairs (not from holotype) type I and III.

**Colour.** Type specimen alcohol faded brown. Live freshly moulted specimens from type locality are an overall blackish with longer red hairs on the abdomen, with grayish hairs on the dorsal trochanter, coxae and edges of the carapace, and two converging stripes on patella in older specimens (Fig. 2). These colours fade to overall brown with subdued russet abdominal hairs after a few months and the first dry season (RG pers.obs.).

##### Distribution.

Only known from type locality Barro Colorado Island, = Lake Gatun/ Canal Zone, Districto La Chorrera, Provincia de Panamá, República de Panamá [DMS = 9°09'00"N, 79°50'41"W].

##### Remarks.

Originally, this species was described by “Carapace is decidedly longer than wide. Median depression transverse; deep” and “barely a trace of scopula on metatarsus IV”. Our examination confirmed these features, but lead us to conclude identification as *Sericopelma* as defined here, including presence of an apically swollen unilobar spermathecae (Fig. 1, see also Figs 3–5, 7–9, contrast 13–16). The type locality of Barro Colorado Island is the site of a Smithsonian Institute field-centre; hence there is a large series of specimens from type locality assignable to *Sericopelma embrithes* (Fig. 2) in the MCZ, MIUP and PMY (supplementary material). It is possible that *Sericopelma embrithes* (Chamberlin and Ivie 1936) is a junior synonym of another *Sericopelma* sp. such as *Sericopelma commune* Pickard-Cambridge, 1897 or *Sericopelma panamanum* (Karsch, 1880). Unfortunately, the mature male of *Sericopelma embrithes* remains unknown. However, geographic considerations can be vital to make confident decisions about both generic and species identities as many tarantulas have narrow distributions, and we contend these older named Panamanian species were collected in distant western Panama, namely ‘Chiriquí’, likely the cool highlands near Volcán Baru and Boquete (Prov. de Chiriquí) where Europeans would acclimatize (rather than the small modern village of Chiriquí, Prov. de Chiriquí). Conversely, *Sericopelma embrithes* from Barro Colorado Island (Prov. de Panamá) is within the central Canal Zone, a distance of over 300 km from ‘Chiriquí’ (Specifically *ca.* 320 km from Panama City to Boquete).

**Figure 6–11.**
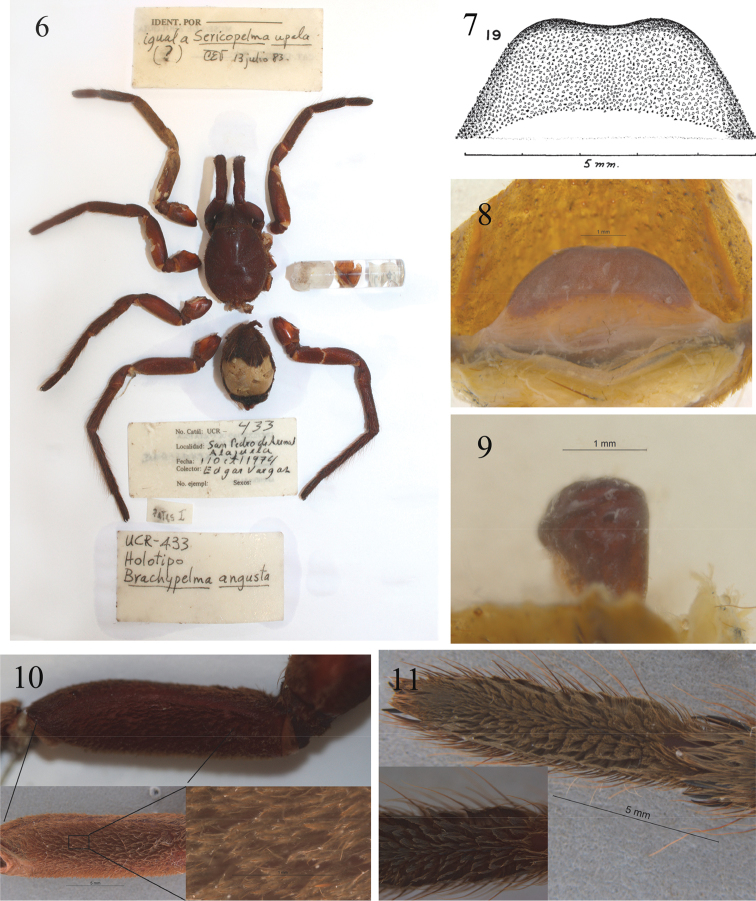
Holotype of *Sericopelma angustum*. **6** Habitus and labels **7** Valerio (1980) figure 19, drawing spermathecae **8** Spermathecae, dorsal view **9** Spermathecaee, lateral view showing (reversed) ‘P-shape’ diagnostic of *Sericopelma* **10** Dense pad of plumose hairs on femur IV not present in *Brachypelma*, upper with alcohol wet, bottom left inset same dried, bottom right inset closeup of plumose hairs **11** tarsus leg IV showing unusual spines along central axis, bottom left inset closer image.

#### Sericopelma angustum

Taxon classificationAnimaliaAraneaeTheraphosidae

(Valerio, 1980) comb. n.

Brachypelma angusta Valerio, 1980: 269, f. 19. (D female)
Euathlus angustus : Raven 1985: 150 (T f from *Brachypelma*).
Brachypelma angustum : Schmidt 1992: 10, f. 8 (T f from *Euathlus*).
Brachypelma angustum : Schmidt 1993: 82, f. 192. (misidentification*) [*Note: The figure ‘Abb. 192’ in Schmidt 1993 shows a spermathecae of an alleged *Brachypelma angustum*, but does not conform to either the Valerio’s (1980) drawing of the holotype spermathecae, nor our examination of the type. We suggest the material of Schmidt (1993) was likely misidentified pet trade *Brachypelma* sp. as with discussion and figures in Peters (2000, 2003), also misidentified pet trade *Brachypelma* sp.]

##### Description.

*Female* (Holotype UCR 433): Total length including chelicerae 58.9. Carapace, length 22.9, width 19.2.Caput, high. Ocular tubercle, length 2.6, width 3.1. Anterior row procurved, posterior row recurved. Eyes, ALE > PLE, PLE > AME, AME > PME. Clypeus, 0.5, clypeal fringe long. Fovea, deep transverse. Maxillae, with 80–100 cuspules, covering approximately 60% of proximal edge. Labium, length 2.9, width 3.7, with 21 labial cuspules (a bald area in the centre of the labium lacks sockets for cuspules and may indicate previous damage, this cannot be confirmed until further specimens are examined) most separated by less than 0.5–1 times the width of a single cuspule. Labio-sternal mounds separate. Sternum damaged, narrow, length 10.2 (approx), width 8.4 with three pairs of sigilla. Femur IV with a dense pad of plumose hair on retro-lateral surface, pro-lateral surfaces of trochanter/femur of anterior legs lacking stridulatory setae. Tarsi I–IV densely scopulate, tarsus IV with spines along central axis. Metatarsal scopulae, I 84%, II 78%, III 35%, of the length of the segment, IV lacking scopulae. Lengths of leg and palpal segments see Table 1. Spination: femurs I, II, IV d 0-0-1, III 0-0-4, palp 0-0-2 (no spines on LHS palp only on RHS palp), patella II, palp 0-1-0, III 1-1-0, tibia I d 0-2-0, v 4-3-3, II d 1-1-1, v 2-4-3, III d 2-2-2, v 3-5-3, tibia IV d 2-0-4, v 4-4-3, palpal tibia d 0-2-1, v 2-2-4 (apical), metatarsus I v 2-0-3, II d 0-1-1, v 2-1-3(apical), III d 3-3-2, v 3-5-10 (6 apical), IV d 6-5-4, v 8-11-16 (6 apical). Posterior lateral spinnerets with three segments, basal 3.9, medial 3.2, digitiform apical 5.1.Lateral median spinnerets with one segment. Spermathecae, single domed receptacle apically swollen with slight medial indentation. Urticating hairs, type I and type III present.

**Table 1.** *Sericopelma embrithes* female holotype lengths of legs and palp.

	I	II	III	IV	Palp
Femur	17.9	17.5	14.8	19.9	14.5
Patella	10.8	9.7	8.7	10.6	9.0
Tibia	12.7	13.2	11.3	15.5	9.8
Metatarsus	12.7	12.2	14.6	21.3	-
Tarsus	9.9	9.8	9.8	11.0	11.1
Total	64.0	62.4	59.2	78.3	44.4

**Table 2.** *Sericopelma angustum* female holotype lengths of legs and palp.

	I	II	III	IV	Palp
Femur	16.3	15.2	14.6	18.9	11.9
Patella	9.5	8.9	8.3	9.7	7.1
Tibia	14.0	12.0	11.2	15.1	9.4
Metatarsus	12.0	12.0	15.0	22.0	-
Tarsus	9.6	9.4	9.1	9.6	9.8
Total	61.4	57.5	58.2	75.3	38.2

**Colour.** Alcohol faded brown, posterior legs III and IV with longer reddish setae.

##### Distribution.

Only known from type locality San Pedro de Arenal, Cantón San Carlos, Provincia de Alajuela, Costa Rica. [Likely DMS = 10°22'30"N, 84°34'47"W].

##### Remarks.

The holotype is now fragmented (Figs 6–11) and right legs II and III both appear to have been lost in life as coxal stumps are blackened indicating wound healing. Accession data from UCR and jar labels specify the holotype was collected on 01-Oct.-1974 by Edgar Vargas, but this information was not given by Valerio 1980. In the holotype jar of *Sericopelma angustum* a label “iqual a *Sericopelma upala* (?) CEV 13 julio 83” (Fig. 6) shows Valerio himself (= CEV) had doubts about placement in *Brachypelma*, also considering it conspecific to the male he described as *Sericopelma upala*. The type localities are close, less than 50 km apart in Alajuela with similar ecotypes of lowland tropical forest, now largely fragmented to sugarcane plantation and cattle pasture (S. Longhorn pers. obs). However, until further specimens of *Sericopelma upala* and/or *Sericopelma angustum* are examined, we are not prepared to place them into synonymy at this time. We suspect Valerio (1980) lacked sufficient access to *Brachypelma* material to make a more informed decision about the genus, failing to recognise defining characteristics (as outlined below).

#### Sericopelma commue

Taxon classificationAnimaliaAraneaeTheraphosidae

F.O.P.-Cambridge, 1897

Sericopelma communis F.O.P-Cambridge: 15 (D male).
Sericopelma commune Smith, 1991b: 18 (f), here considered misplaced in this species.

##### Type.

*Male* (3 male syntypes, BMNH 1898-12-24-19-21, male syntype OUMNH O.P.-Cambridge Coll. Jar 106):

##### Remarks.

Smith (1991b) refers to three of four male syntypes from Chiriquí as *Sericopelma commune*, specifically BMNH 1898-12-24-19-21 (*i.e.* accessioned 24^th^-Dec-1898, coded ‘19-21’), then described a female, saying “Female BMNH 98-12-24-22. Assigned to the species by Valerio”. The only female BMNH specimen with this accession has the oldest label “*Museo Nacional de Costa Rica, Pozo Azul de Pirrís, José C. Zeldón*”, naming a collector from the 1890s. A later label “*Sericopelma immensa* n. sp. Det. C. E. Valerio, Jan 10, 1979” matches his paper (Valerio 1980) referring to a BMNH specimen from this same locality as *Sericopelma immensum*. However, the species on the Valerio label has been physically scored out, but likely reads *immensa*. Another pen-written label says “*Sericopelma commune* F.O Pick–Cambr.” (in handwriting of curator Doug Clark, died 1972), apparently present when both Valerio and Smith examined the specimen. We suspect this label misled Smith (1991b) to reconsider the specimen as the un-described female *Sericopelma commune*, even though collected at a Costa Rican locality (Parrita Cantón, Puntarenas), approx. 250 km from the Chiriquí type site. However Smith only records the distribution (indicating both sexes) from Chiriquí, Panama. Further confusion occurs with another mature male in BMNH with an old pencil-written label “Panama”, then two pen labels in Clark’s handwriting, “*Sericopelma commune* PDA Costa Rica BMNH 1898-12-24-22” and “*Sericopelma commune* det. Clark 1960”. We suspect these latter labels were an attempt by Clark to wrongly allocate this “Panama” male to both the Pozo Azul de Pirrís accession, and as a ‘missing’ fourth male syntype of *Sericopelma commune*. Clark perhaps did not realise that fourth male is in the Pickard-Cambridge collection at OUMNH, where a male labelled ‘syntype’ had the unequivocal label “*Sericopelma communis* Fopc Chiriqui – Champion”. In a BMNH accessions book, 1898-12-24-22 corresponds to “*Sericopelma* sp? Pozo Azul de Pirrís (Costa Rica). Pres. by F.D. Godman, Esq., Costa Rica Mus, F.O.P.-Cambridge”. However, although F.O. Pickard-Cambridge apparently recognised it as a possible female *Sericopelma* sp, the lack of accounts before Valerio (1980) indicate it was ignored, perhaps due to uncertainty about matching it with known males. We consider this female to be the same listed by both Valerio (1980) and Smith (1991b) and suggest its unsecure designation as the first described female of *Sericopelma commune* be suspended, instead to favour topotypic specimens from Chiriquí, such as the region of Volcán where G. Champion likely collected the four male syntypes.

##### Distribution.

Only known from type locality, Chiriquí = Chiriquí, Provincia de Chiriquí, República de Panamá.

#### Sericopelma panamanum

Taxon classificationAnimaliaAraneaeTheraphosidae

(Karsch, 1880) stat. rev.

Theraphosa panamana Karsch, 1880: 84 (D male).
Sericopelma panamana F. O. Pickard-Cambridge, 1897a: 16.
Sericopelma rubronitens Simon, 1892: 159 (S, here considered misplaced in this species).

##### Type.

*Male* (1 male holotype, ZMB 2394 BERLIN):

##### Remarks.

Simon (1892) makes no clear justification why Karsh’s *Theraphosa panamana* from Chiriquí should be synonymous with *Sericopelma rubronitens*, only referring to similarities in eye pattern and absence of tibial spurs in Karsh’s description against another non-type male specimen in the Paris collection, which he had assigned as *Sericopelma rubronitens*. Our re-examination of the type specimen confirmed its designation as a *Sericopelma* sp., but not its synonymy with *Sericopelma rubronitens*, which is here reversed.

##### Distribution.

Only known from type locality, Chiriquí = Chiriquí, Provincia de Chiriquí, República de Panamá.

### Geographic distribution, and generic limits

We believe it is important to re-clarify the characteristics of *Brachypelma* in this context. The type of *Brachypelma* is *Brachypelma emilia*, originally suggested in the paper’s title to be from Panama (White 1856). A later male from México: Ventanas, Prov. de Durango (leg. Forrier) was described by Simon (1891) as generic type. Smith (1994) incorrectly says “Simon lists his specimen as coming from Panama” (p.166). We suspect this stems from a mis-listing by F.O.P-Cambridge (1897) of “PANAMA (coll. Simon: Male)” where locality was confused with the original type. While the original specimen appears lost (Smith 1994) or ‘non-existent’ (Pickard-Cambridge 1897), an excellent illustration in White’s paper allows identification, showing an adult male with tibial spurs. The route of the collector (Berthold Seemann) is well known (Seemann 1852), joining his ship in Panama and voyaging north along the Pacific, docking in México both at San Blas (Estado Nayarit) and Mazatlán (Sinaloa). The original type taken to the BMNH was most likely collected during the second inland foray in 1849/50 to Ciudad de Durango (modern Victoria de Durango, Durango) and Tepic (Nayarit). However, another male deposited in MNHN was used as generic type of *Brachypelma*, from Ventanas (leg. Forrer). This is likely modern Villa Corona, Estado Durango (DMS = 23°52'51"N, 105°46'19"W) (Selander and Vaurie 1962), but concurs both with the route of Seemann (within 15 km from Mazatlán to Ciudad de Durango) and with modern understanding of the species distribution across Sinaloa, Durango and Nayarit (Locht et al. 1999). Pickard-Cambridge (1897) mentions two males by Mr. Forrer from Ciudad [modern Victoria de Durango] plus Simon’s male from Ventanas, but none specifically as neotype. One adult male which Smith (1994) refers to as neotype was accessioned in NHM as BMNH 98-12-24-32 where it is labelled ‘leg. Forrer’ plus ‘Ciudad’. The second adult male is in the Pickard-Cambridge collection at OUNMH (Jar 65), with the same collection details of ‘Ciudad. Mex, Forrer’, plus labelled ‘paratype A.M. Smith’. However, we argue preference could have been given to the generic type of Simon from Ventanas. Simon (1891) also referred to a female specimen, though Pickard-Cambridge (1897) stated the female is unknown. However Smith (1994) gives a comprehensive description of both sexes, using a later female BMNH 1962-2-28-1, and as a result the taxonomic identity of this species is clear. Simon (1891) originally emphasized several characters for *Brachypelma*, including presence of distinct scopula on the metatarsus, and femur IV without inner scopula (i.e. no dense pad of plumose hairs), instead long and simple hairs (“*metatarsus paris scopula crassa medium articulum fere attingente munitus, femora postica haud scopulata intus longe et simpliciter pilosa*”, Simon 1890). The genus is also characterised by plumose hairs on the prolateral face of leg I trochanter/femur and retrolateral face of the palp (Pocock 1903). These features have been supported by subsequent authors as diagnostic for *Brachypelma* (*e.g.* Smith 1994), such as both sexes without a plumose pad on leg IV femur, the metatarsus IV distally one-third to one-fifth scopulate, and no tarsal division by stiffened setae, along with male palpal bulb distally wide and flattened (spoon-shaped), two unequal spurs on male tibia of leg I, females with a simple undivided/fused spermathecae (Figs 13–14) which we further clarify have a flat cross-section. Despite some earlier confusion about the types, the type species *Brachypelma emilia* is well defined, and the genus is easily separated from *Sericopelma*. The geographic range of *Brachypelma* is securely centred in south-western Mexico, now with *Brachypelma albopilosum* and *Brachypelma fossorium* at its southern-most limit in Northern Costa Rica. Due to the generic transfers here of *Sericopelma angustum* and *Sericopelma embrithes* (and comments below on other specimens), there are now no reliable records of the genus *Brachypelma* in Panama. The transfers proposed here verify that the *Brachypelma* as currently defined ranges from Mexico to north Costa Rica, and is not native in Panama or further south.

**Figure 12.**
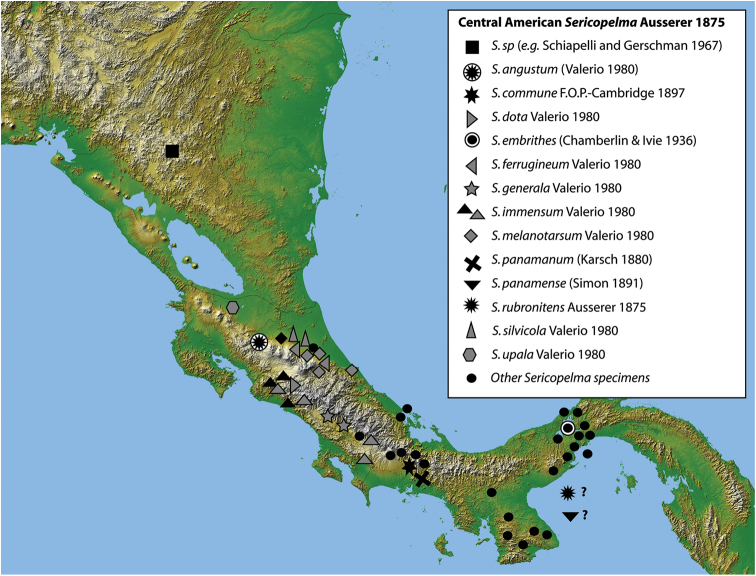
Geographic distribution of the genus *Sericopelma* from published records (including this study), where complete black-centred shapes are for specimens examined during this study, whilst gray shapes [outlined in black] are further specimens listed by Valerio (1980), accordingly data for *Sericopelma immensum* has black shapes (for the holotype, allotype and further female from Pozo Azul de Pirrís examined here), and gray shapes for further sites of Valerio. *Sericopelma rubronitens* and *Sericopelma panamense* are of unspecific location, but canal-zone seems likely.

**Figure 13–16.**
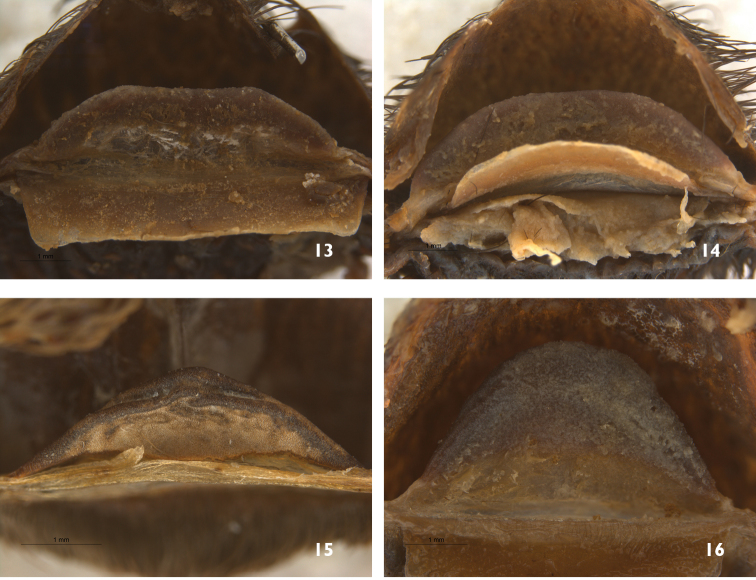
Selected taxa with similar spermathecae to *Sericopelma*. **13** *Brachypelma emilia*, type species of the genus from México, specimen EME10 in SJLC **14** *Brachypelma verdezi* from México, PAL4 in SJLC **15** *Megaphobema robustum* type species of the genus from Colombia, OUMNH 2008 072 (ROB3); and **16** *Megaphobema mesomelas* from Costa Rica as MES4 in SJLC.

**Geographic distribution of *Sericopelma*.** From examination of specimens (see methods and supplement), combined with data we consider reliable in Schiapelli and Gerschman (1967) and Valerio (1980), we consider that *Sericopelma* ranges from Nicaragua to Panama (Fig. 2), with the northern-most report from Nicaragua. This was confirmed by examination of a single male specimen from Matagalpa, Nicaragua held in MCZ.

We regard the inclusion of 'Guatemala' in the original type locality of *Sericopelma panamense* from 'Panama, Guatemala' as an error, and suggest that 'Guatemala' instead refers to the locality for a second specimen (actually from another genus, and seemingly not of a taxon from Panama) which we found in the same jar from the Paris collection.

**Panama [Provincia**]: *Sericopelma commune* Pickard-Cambridge 1897 [Chiriquí]; *Sericopelma embrithes* (Chamberlin & Ivie, 1936) [Panamá]; *Sericopelma panamense* (Simon 1891) [Unspecified*]; *Sericopelma rubronitens* Ausserer 1875 [Unspecified**] (including as junior synonym *Sericopelma panamanum* (Karsch 1880) [Chiriquí]). **Costa Rica [Provincia**]: *Sericopelma angustum* (Valerio 1980) [Alajuela]; *Sericopelma dota* Valerio 1980 [San José]; *Sericopelma ferrugineum* Valerio 1980 [Cartiago, Heredia]; *Sericopelma generala* Valerio 1980 [San José]; *Sericopelma immensum* Valerio 1980 [San José, Puntarenas]; *Sericopelma melanotarsum* Valerio 1980 [Alajuela, Cartiago, Heredia; Limón]; *Sericopelma silvicola* Valerio 1980 [Cartiago, Heredia, Limón]; *Sericopelma upala* Valerio 1980 [Alajuela, Cartiago]. **Nicaragua [Departmento**]: *Sericopelma* sp. indet. [Matagalpa] (*e.g.* Schiapelli and Gerschman 1967).

*Note*: The extralimital Brazilian *Sericopelma fallax* Mello-Leitão, 1923 is considered misplaced (see Gabriel and Longhorn 2011). * Originally listed as Panama and Guatemala, though the latter is unlikely. ** Originally simply listed as Panama.

## Discussion

Prior to Valerio (1980) the diagnostic features for *Sericopelma* were poorly known, with males primarily recognised by the palpal bulb shape and absence of tibial apophyses (Ausserer 1875, Karsch 1880, Simon 1891/82), while females were unrecognized until Schiapelli and Gerschman (1967). Over-reliance on the lack of male tibial apophyses led many museum specimens to be mislabelled and misplaced. In actuality, Simon (1891) had described the first female *Sericopelma* as *Eurypelma panamense*, but unrecognized until Gabriel (2009) rediscovered it as a former *Eurypelma*, a genus that Raven (1985) had described as a taxonomic “dumping ground”. We now confirm that Chamberlin and Ivie (1936) misplaced another female into *Eurypelma*, here transferred to *Sericopelma embrithes* (Chamberlin & Ivie, 1936). As the female characteristics of *Sericopelma* have long been uncertain, the female description by Smith (1991b) was valuable to resolve uncertainty about spermathecae characteristics. Schiapelli and Gerschman (1967) illustrated the first spermatheca of a probable *Sericopelma* from Nicaragua (Fig. 3) [Nb. specimen not seen]. Their relatively poor illustration shows possible indentations or notches on the apex, which appears atypical of the genus. However, we confirm that *Sericopelma* indeed exists in that region from another examined male *Sericopelma* sp. in MCZ with the label “Matagalpa, Nicaragua”. Valerio (1980) described seven species from Costa Rica, only illustrating the spermathecae of both *Sericopelma immensum* and *Sericopelma silvicola* as simple domes, and neither shows any such notches. Neither do spermathecae of Smith (1991b) nor Schmidt (1994) show any such notches. Perez-Miles et al. (1996) reproduced the Schiapelli and Gerschman (1967) illustration, stating female *Sericopelma* have “a single spermathecae receptaculum with a median notch”, plus key “19. Female with notched spermathecae”. Schmidt (2003) also referred to the *Sericopelma* spermathecae as “*Einteilige flache*” (*i.e.* single flat) using the same illustration, not mentioning any apical notches or indentations. We regard the ‘notched spermathecae’ of Schiapelli and Gerschman (1967) as misleading, and its use to define female *Sericopelma* as erroneous. We find that mature female *Sericopelma* spermathecae lack any distinct median notch (Fig. 4) and furthermore, are distinctly swollen on the apex producing a diagnostic P-shape when viewed in profile (Fig. 5), which is also diagnostic for most immature *Sericopelma* females. We suggest this apical swelling probably expands with age (*i.e.* ontogenetic modification). Although the holotype spermathecae of *Sericopelma angustum* does have a slight medial concaved indentation, we consider this unique. It also shows the diagnostic swollen apex with P-shaped profile diagnostic for *Sericopelma*. The swollen apex is not found in the other Neotropical theraphosid genera where females have a single unilobar spermathecae, instead flattened or apically narrowed cross-section, such as *Brachypelma* Simon, 1890, *Megaphobema* Pocock, 1901 and *Theraphosa* Thorell, 1870. Female *Sericopelma* can be distinguished from *Eupalaestrus* Pocock, 1901, *Vitalius* Lucas, Silva & Bertani, 1993, *Nhandu* Lucas, 1983, *Pamphobeteus* Pocock, 1901 and *Xenesthis* Simon, 1891 by the unilobar spermathecae lacking two separated apical projections (Bertani 2001), and from *Mygalarachnae* Ausserer, 1871, by the unilobar structure lacking a broad median notch (Gabriel and Longhorn 2011).

**Figure 17–18.**
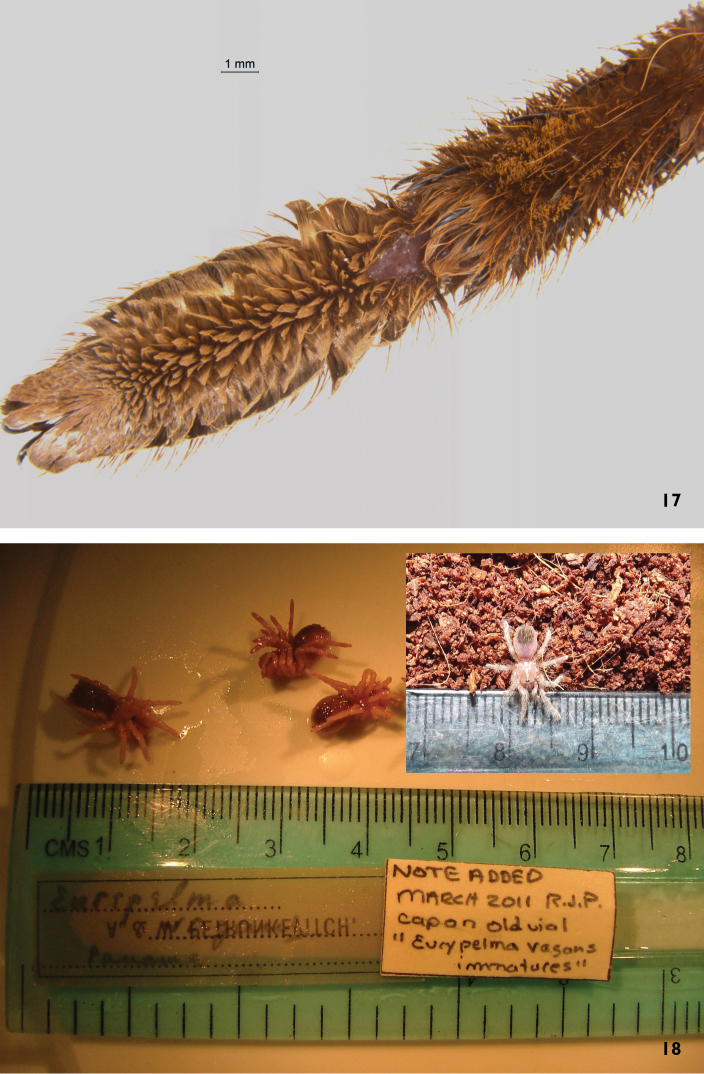
**17** Leg IV tarsus and metatarsus of *Sericopelma immensum*, allotype female (Naranjal de Guarumal, Cantón Puriscal, San José, Costa Rica), showing most extensive metatarsal ‘trace’ scopula **18** Nymphal (pre-dispersal) young misidentified by Petrunkevitch as *Brachypelma vagans* Panama, and inset, older yet smaller (post-dispersal) young of Brachypelma cf. vagans (pettrade, from Mexico).

Along with spermathecae attributes, *Sericopelma* can now be defined by; Carapace longer than wide (Ausserer 1875, Karsch 1880, Simon 1892, Pickard-Cambridge 1897, Schiapelli and Gerschman 1967), deep transverse fovea (Ausserer 1875, Karsch 1880, Pickard-Cambridge 1897) and distinct radiating sulci (Ausserer 1875). We confirm these attributes as useful for both sexes, although carapace is more rounded in mature males than females. Another useful diagnostic is few/weak metatarsal scopulae on distal leg IV forming two distinct pads, elsewhere defined as “barely a trace of scopula on metatarsus IV” (Chamberlin and Ivie 1936), “not scopulate, or very slightly so at the apex” (Pickard-Cambridge 1897), or absent (Ausserer 1875, Simon 1892, Valerio 1980, Schiapelli and Gerschman 1967). Here we confirm that almost every examined specimen of *Sericopelma* actually does have trace of scopulae on the distal leg IV metatarsus, most forming two small distinct pads when viewed ventrally (Fig. 17, in most extensive form). Such ‘trace scopulae’ are typically present on in both mature sexes, but in some specimens are distinct while in others greatly reduced. The fresh specimens that lacked trace scopulae were smaller juveniles, suggesting the feature may become more conspicuous through development. Trace scopulae were absent on some larger specimens, but only when eroded through wear or damage. Our examination of *Sericopelma angustum* confirmed trace scopulae on leg IV metatarsus as with other *Sericopelma*, unlike the one-third to one-fifth scopulae present in *Brachypelma*. From a large array of specimens (see Supplement), female *Sericopelma* may be robustly defined by: Spermathecae single (unilobar), swollen at the apex to form a P-shaped cross-section, femur IV with a dense retrolateral pad of plumose hair, trochanter/femur of leg I lacking stridulatory setae, carapace longer than wide, deep transverse fovea and distinct radiating sulci, ventral metatarsus IV with a divided and reduced trace of scopulate hairs at the distal end. Apart from spermathecae attributes, these remaining features also define mature males along with the absence of tibial spurs and characteristic embolus shape.

The dense retrolateral pad of plumose hair on femur IV is another useful character to separate *Sericopelma* from *Brachypelma*. We clarify the term ‘femoral scopula/e’ in *Sericopelma* as a broad pad of plumose hairs. Valerio (1980) defined *Brachypelma* with “Scopula in femur IV inconspicuous or absent”, as did subsequent authors (Smith 1994, Schmidt 2003). Yet Valerio (1980) had previously confirmed that femur IV of *Brachypelma angustum* does indeed have a modified patch of hairs, by “*Femur IV con cojinete medial*” (p. 270), and elsewhere confirmed *Sericopelma* indeed posses such. Our examination of the *Sericopelma angustum* holotype (Figs 6–11) showed a broad pad of plumose hair on retrolateral femur IV (Fig. 10) as in other *Sericopelma* spp., but not *Brachypelma*. Schmidt and Krause (1994) reported that *Brachypelma klaasi* is exceptional with a “thin pad of plumose hairs on femur IV”, used to support a new genus *Brachypelmides*, since rejected. They gave no indication of which sex was examined nor where femoral hairs were found. We therefore also examined mature *Brachypelma klaasi* specimens of both sexes and found no distinct pad on retrolateral femur IV, just a few sporadic fine-hairs slightly plumose basally, near the distal femur. We suggest these conform to the diagnostic ‘short weak-feathered hairs (= kurze schwachgefiederte Haare) of Schmidt and Krause (1994), but do not form any distinctive pad as in *Sericopelma* (as *Sericopelma angustum* and *Sericopelma embrithes*). Instead in *Brachypelma klaasi*, these modified hairs are interspersed among more numerous long-fine hairs and thicker bristle-like hairs. Further, there is a bald-line forming a longitudinal strip along the axis in *Brachypelma klaasi*, observable in both fresh and alcohol preserved specimens, contrasting with the dense pad of plumose hairs in *Sericopelma*. Modified hairs of *Brachypelma klaasi* hind-femurs were difficult to distinguish on alcohol-preserved specimens, so we also examined dried exuvia as Schmidt and Krause (1994), where fine-basally plumose hairs were more easily detected. Other examined *Brachypelma* spp. only showed fine hairs and bristle like hairs on femur IV, as reported for *Brachypelma albiceps* by Locht et al. (1999).

With a more robust definition of *Sericopelma* (including female characteristics), we can be increasingly certain about generic boundaries. Valerio (1980) defined *Sericopelma* by “the presence of a thick scopula in the inner side of femur IV, the absence of spurs on tibia I, [absence of] stridulatory setae on trochanter I, and [absence of] scopula on metatarsus IV”. Also “One spermathecae, semicircular, sometimes with lateral extensions, covered with fine spinules”, or as “*Receptaculum seminis* opens on dorsal side of apical region, communicating with distal tip of bulb by and open groove.” This may be alluding to the apically swollen P-shaped cross-section that we consider diagnostic for *Sericopelma*. Valerio appears to have been misled by the central depression he characterised as “Spermathecae with a shallow notch in anterior edge (Fig. 7 [his figure 19])”, leading him to recognise similarity with *Brachypelma albopilosum*, and misdiagnosing them both as *Brachypelma* by shared “Spermathecae with a conspicuous depression on the anterior edge”. Our examination of *Sericopelma angustum* showed the spermathecae indeed possesses a slight medial indentation, but less defined than Valerio suggested, and we further recognise the apical swelling with a P-shape cross-section (Figs 8, 9) as diagnostic of *Sericopelma*. Spermathecae of other genera like *Brachypelma* (Figs 13–16) are flat throughout in cross-section. Further, *Sericopelma angustum* does not have any plumose hairs on the prolateral trochanter or femur of leg I (or II), nor the retrolateral palpal trochanter (*i.e.* Smith 1994, Schmidt 2003), but does have a distinctive pad of plumose hairs on femur IV (Fig. 10), together confirming it as *Sericopelma*, representing a unique species due in part to distinctive spines on tarsus IV (Fig. 11).

During this study, we found many historical museum specimens with mistaken identities, most importantly several wrongly reported as Panamanian *Brachypelma*. Petrunkevitch (1925) listed *Sericopelma commune*, 1 male and 1 female from the Canal zone. *Sericopelma rubronitens* from 2 females from Culebra (probably Pacific Canal Zone, ‘Gaillard Cut’), and 2 females from Bocas del Toro. As discussed above, *Sericopelma commune* was described from males collected in distant Chiriquí, hence the identity of his Canal Zone species is dubious. Petrunkevitch did not compare his specimens to the earlier male types (nor could he with females), so his determination of various females as *Sericopelma rubronitens* cannot be regarded as reliable descriptions. Our confidence in Petrunkevitch determinations is greatly reduced as he also misidentified other geographically diverse specimens as *Sericopelma rubronitens*, all from outside the geographic range of the genus *Sericopelma*, such as from México, Haiti, and Ecuador (see supplement for re-evaluation), probably as all were similarly coloured with dark bodies and reddish abdominal hairs. He also inconsistently referred to specimens from Barro Colorado Island as either *Sericopelma rubronitens* or *Sericopelma commune* (see supplement), despite being the type locality for *Sericopelma embrithes*. Petrunkevitch (1925) mistakenly reported several *Brachypelma* from Panama, namely *Brachypelma emilia*, 1 female of *Brachypelma sabulosum* from Culebra, 1 female of *Brachypelma vagans* from Culebra, plus 4 young *Brachypelma vagans* specimens without locality. For *Brachypelma emilia*, Petrunkevitch (1925) merely repeated the erroneous location from the original description. Interestingly, some male *Sericopelma* from Chiriquí do superficially resemble *Brachypelma emilia* by light pinkish lower legs and carapace, plus black triangle on the carapace, perhaps leading to early confusion. On re-examination of the Petrunkevitch specimens in PMY, his alleged *Brachypelma sabulosum* was a *Sericopelma* sp, as likely are the 4 immatures of alleged *Brachypelma vagans*. The immatures are pre-dispersal nymphs, with the wrong proportions for *Brachypelma vagans* – where nymphs are almost one fifth of this size. In *Brachypelma vagans*, the legs remain proportionally shorter even when older post-dispersal ‘spiderlings’ of equivalent size (Fig. 18). The most likely genus for these large nymphs is *Sericopelma*. The alleged female *Brachypelma vagans* was not located, but we also expect to be a misidentified *Sericopelma*, which can be similarly coloured and often confused by non-specialists. Distribution of *Brachypelma sabulosum* and *Brachypelma vagans* from Panama should be regarded as mistaken, *Brachypelma sabulosum* is only validly recorded from Guatemala, whilst *Brachypelma vagans* is recorded from México, Belize and Guatemala.

Finally, another allied Costa Rican species with long ambiguous placement is *Megaphobema mesomelas* (O.P.-Cambridge, 1892), again originally placed in the poorly defined *Eurypelma*. Valerio (1980) described the first female and transferred it to *Brachypelma* before Schmidt (1991a/b) transferred to *Megaphobema*. Smith (1991b) also re-evaluated the species, drawing tarsus IV with twin central lines of modified setae (his figure 6), which Valerio had recognised as “*Cojinete del tarso IV dividido por varias filas de espinas*”. Against this, we considered *Sericopelma angustum* where scopulae are interspersed by thickened spines (Fig. 11), which we consider species specific - as not observed in other *Sericopelma*, nor mature specimens of other candidate genera. However, our inspection of various recent (both sexes) and historical specimens of *Megaphobema mesomelas* (including the male holotype and another male from same collector in the O.P.-Cambridge collection), each revealed only few long soft hairs on tarsus IV, not thickened spines. Our re-examination of *Megaphobema mesomelas* lead us to agree it does not belong in *Brachypelma*, nor *Sericopelma*, but neither do we agree with placement in *Megaphobema* (Gabriel and Longhorn, in prep). Female *Sericopelma* can be distinguished from *Megaphobema* by the form of the spermathecae, in the latter by greater ventral surface sculpturation with striated grooves more evenly spaced and extending to lateral edges, or a more cerebriform pattern, plus flatter cross-section (Fig. 15). Mature males of *Sericopelma* lack tibial apophyses (as do some other genera), but are present in *Megaphobema* (and other genera). Both sexes of *Megaphobema* also can be distinguished from *Sericopelma* by more extensive scopulae on metatarsus IV. For *Megaphobema mesomelas* the sternum is especially narrow and elongate, which Smith (1987) says “over twice as long as wide”. We agree, observing the *Megaphobema mesomelas* sternum is more extremely narrowed than *Sericopelma angustum*. The narrowed form in both conflicts with *Brachypelma*, defined by a broad sternum (*i.e.* Simon 1891, “*Sternum aeque longum ae latum*”). *Sericopelma angustum* was diagnosed by Valerio (1980) by “Carapace longer than 18.0 mm” or “Carapace very narrow (1.6 times longer than broad)”, and his specific epithet ‘*angust*’ (= narrow) refers to both the narrow cephalothorax and sternum. We suggest the narrowed sternum can be indicative of close evolutionary affinities of *Megaphobema mesomelas* with *Sericopelma*, particularly *Sericopelma angustum*.

### Consequences for conservation, including CITES

Currently, all *Brachypelma* species are protected by international commercial trade regulation (CITES, Appendix II). Transfer of *Sericopelma embrithes* and *Sericopelma angustum* into *Sericopelma* means that consequently these species may now only be protected by national wildlife laws. However, there does not appear to be a current need to regulate trade in *Sericopelma embrithes* and *Sericopelma angustum*, so we assert both species should indeed be removed from CITES listing. As with most theraphosids, the major threat appears to be habitat destruction. For *Sericopelma angustum*, much of its probable habitat in northern Costa Rica has already been disrupted by human activity, often for sugar cane plantations. However, its conservation status within Costa Rica must be urgently evaluated. For *Sericopelma embrithes*, much of its original range was likely destroyed during the damming of the Chagres River for the Panama canal, isolating Barro Colorado Island. A more deserving candidate for CITES regulation is *Megaphobema mesomelas*; a large brightly coloured species which has regularly been targeted by illegal collection for commercial gain, and traded internationally. We also point out there remains need for continued regulation of all *Brachypelma* sp. traded as exotic pets, including those in the pet-markets still exchanged under the former name ‘*Brachypelma angustum*’, which would retain their CITES protected status under the aegis of *Brachypelma* sp.

## Supplementary Material

XML Treatment for Sericopelma embrithes

XML Treatment for Sericopelma angustum

XML Treatment for Sericopelma commue

XML Treatment for Sericopelma panamanum

## Appendix

Additional non-type material for comparison. Note – SJLC are mainly pet-trade, often of unclear providence unless otherwise stated, plus exuvia of other immatures and adults]

***Sericopelma* specimens**:

***Sericopelma embrithes*** [***Panama, Canal zone = Prov. Colón, Panamá and Panamá Oeste*] - MCZ**: 2 ♀ 2 Imm, *Sericopelma embrithes* 74344 July 1936, B.C.I; 1 Imm ♂*Sericopelma embrithes* 74335 22/10/1954 B.C.I; 1 Imm ♂*Sericopelma embrithes* 74637 20/10/1950 B.C.I; 1 Imm ♂*Sericopelma embrithes* 74612 7/12/50 B.C.I; 3 Imm ♂*Sericopelma embrithes* 74338 B.C.I; 2 Imm ♂*Sericopelma embrithes* 74345 June – July 1950, B.C.I; 1 Imm ♂*Sericopelma embrithes* 74634 23/07/1950, B.C.I; 1 Imm ♂*Sericopelma embrithes* 74348 06/08/39, B.C.I; 7 Imm *Sericopelma embrithes* unsexed, 74340 16/08/1954, B.C.I; 1 Imm ♀ 1 Imm ♂*Sericopelma embrithes* 74343 B.C.I; 2 Imm ♂ 3 Imm ♀*Sericopelma embrithes* 74342 July 1936 B.C.I; 1 Imm ♀*Sericopelma embrithes* 74635 summer 1939 B.C.I; 1 Imm ♂*Sericopelma embrithes* 74336 summer 1939, B.C.I; 1 ♀ 74373, [no data], 1 Imm ♂ *Sericopelma embrithes* B.C.I., Canal Zone, Sept, coll. Phil Raw; **PMY**: 1 ♀ *Sericopelma embrithes* B.C.I. Canal Zone, Panama, July 1938, Coll. and Ident. (as *Sericopelma rubronitens*) by A. Petrunkevitch, from burrows on the lawn by the laboratory; 2 ♀ *Sericopelma embrithes* B.C.I. Panama, 04.viii.1938. coll. and Ident. (as *Sericopelma rubronitens*) by A. Petrunkevitch, [reverse reads ‘in life with red abdomen]; 1 Imm ♂ *Sericopelma embrithes* (as *Sericopelma rubronitens*) B.C.I. Panama, July 1938, Coll. and Ident. by A. Petrunkevitch [mis-sexed ♀]; 1 ♂ *Sericopelma embrithes* B.C.I. Canal Zone, Panama, Coll. and Ident. (as *Sericopelma communis*) by A. Petrunkevitch, 1 Imm ♂ *Sericopelma embrithes* B.C.I. Panama, July 1938, coll. and Ident. by A. Petrunkevitch, [mis-sexed as ♀];1 ♀ 4 larvae *Sericopelma embrithes* 7 Imm, B.C.I., Canal Zone, A.M. Chickering, 1 ♀ 5 imm *Sericopelma embrithes*, B.C.I., Canal Zone, A.M. Chickering, July 12 1934.

***Sericopelma* spp.** [***Panama, Canal zone = Prov. Colón, Panamá and Panamá Oeste*] - BMNH**: 1 ♀ *Sericopelma* sp. 1926.1.27.14 Taboga Island, Panama, 12/09/24, coll. G.I. Collenette. **MCZ**: 1 Imm ♀ 2 Imm ♂ *Sericopelma* sp. 74341 Ft. Clayton, IPAC. Side Panama, coll. Shropshire; 1 ♂ *Sericopelma* sp. 74346 Fort Davis, C.Z. 1924-1925 coll. Major D.R. Chase; 1 ♂ *Sericopelma* sp.74638 Bouia Point 1927, J. Barbour Don, I.B. Shropshire, collection; 1 ♀ *Sericopelma* sp. Fort Sherman C. Z. [Canal Zone], Feb 1924, det, N. Banks; 2 ♂ *Sericopelma* sp. Ancón, Canal Zone, Mar-April 1922, T. Barbour and W.J. Brooks, det, N. Banks; 1 ♀ 2 ♂ *Sericopelma* sp. Corogae, Canal Zone, Atl.[Atlantic] Side, det. N. Banks. **MUIP**: 1 ♂ *Sericopelma* sp. Panama, Province Panama, Arraijan, San Jose de Bernardino 26.08.2003 coll. M. Barahona; 1 ♂ *Sericopelma* sp. Arraijan Panama 08.1995; 2 ♀ 1 Imm ♂ Vista Bella, Arraijan Panama Rep Panama 08 04 1990 coll. David Maruaga; 2 ♂ *Sericopelma* sp. Huili Arraijan, Panama R.D. Pan 8 November 86 coll. Daniel Holnes; 1 ♀ *Sericopelma* sp. Villalobos, Pedregal. Panama R.D. Pan 05.09.85 coll. Ramito Pinzon, Diana Moreno; 1 ♂ *Sericopelma* sp. Imprenta de la Universidad de Panama. Panamá 17.08.1981 coll. H. Martinez; 1 ♂ *Sericopelma* sp. Ciudad Panama, Republic de Panama, 26.08.1988 coll. Jasmin (sec.esc) Fistica; 1 ♂ *Sericopelma* sp. Panamá province, Panama Campana, L. Ortega 12.09.02; 1 ♂ *Sericopelma* sp. Profomsamilla 13, via Bolivar Panama 16 Oct 1980 coll. Alfonso Chong; 1 ♀ *Sericopelma* sp. Samaria, San Miguelito, Province Panama, Republic of Panama, coll. R. Navarro 16.12.94; 1 ♂ *Sericopelma* sp. Ciudad de Panama, Province Panama, Puerto Nuevo, Republic of Panama, 17.Dic.1977, coll. D. Quintero jnr; 1 ♂ *Sericopelma* sp. Isla Bagano, near bridge wet more, on forest litter, Province Panama, Republic of Panama, 31.01.76, coll. Claudia de Peralta; 1 ♂ *Sericopelma* sp. Province Panamá, Chilibre Carretera Puente de Chilibre, 25m despues del Puente, 15.Oct.1977, coll. D. Quintero jnr; 1 ♂ *Sericopelma* sp. Ciudad de Panama Ivan Diaz de rajo de tronco caido, Republic of Panama, 09.Dec.1977, coll. D. Quintero jnr; 1 ♀ *Sericopelma* sp. Panama, Province Panamá, area del canal, Howard Hecia Ferfan, 7-8.Feb. 2009, coll. S. Ortaga; 1 ♀ *Sericopelma* sp. Republic of Panama, Panamá Oeste Province, Bejuco, Sora, 21.Julio. 2007, coll. R. Carranzo. **PMY**: 1 ♀ *Sericopelma* sp. Culebra, Panama [was misidentified as *Brachypelma sabulosum*]; 4 Imm *Sericopelma* sp. Panama, [were misidentified as *Brachypelma vagans*], 1 ♀ *Sericopelma* sp. Culebra, Panama (described as *Sericopelma rubronitens* in the “Arachnida of Panama”), A. Petrunkevitch; 1 Imm ♂ Sericopelma cf. rubronitens Culebra, Panama; 1 ♀ Sericopelma cf. rubronitens Culebra, Panama, Dr. B.H. Buxton. [***Panama, Central zone = Prov. Coclé, Herrera, Los Santos, Veraguas*] - MCZ**: 1 ♂ *Sericopelma* sp. El Valle, Panama, August 1936 (likely El Valle de Antón, Coclé; 1 ♂ 1 ♀ *Sericopelma* sp. 74371, San Pablo, Panama, Serly (possibly Rio San Pablo, Soná). **MUIP**: 1 ♂ *Sericopelma* sp. Altos de El Valle, Finca el Naranjal, Panama (Coclé), 22 Julio 1979, coll. Elisa De Fuentes; 2 ♂ *Sericopelma* sp. Rio Hato as Pollas Clano Bonito, Farallon el Plastanoc, Las Guias y El Rincon, Coclé, Panama 10.6.85, 24.7.85; 1 ♀ *Sericopelma* sp. Panama, Province Coclé, Río Hato (Las Guias – Farallon), Sept – Oct 1983, coll. A. Parra; 1 Imm ♀ *Sericopelma* sp. Panama, Province Herrera, District Las Minas, R.F. [=Refugio Forestal] El Monteuso - Estacion,13-15.Dic 2002 coll. P. Gonzales; 2 ♀ *Sericopelma* sp. Panama, Province Herrera, R.F. El Montueso, 3 Mayo 2007, coll. R.J. Miranda & A. Santor; 2 ♂ *Sericopelma* sp. Theraphosidae *Sericopelma commune* F.O.P.C. 1897 Det R.J. Miranda 2004 Panama, Province Los Santos, Cerro Canajagua 830m alt 11.12.02 coll. D. Gonzalez; 1 ♂ *Sericopelma* sp. Panama, Prov. Los Santos, Honantial 19. Augusto. 2000 L. Shamix; 1 ♂ *Sericopelma* sp. Panama, Province Herrera, San José, Oct 24 de Leo 1989, coll. K.F. Ponce; 1 ♀ *Sericopelma* sp. Panama Province Los Santos, R.F. La Trondsa, La Trondsa, 14-17 Augusto 2007, coll. R.J. Miranda. 1 ♂ *Sericopelma* sp. San Juan, Rio Cañazas, Province Veraguas, Panama, 22.Agusto.1987, coll. D. Quintero. [***Panama, West Zone = Prov. Chiriquí, Bocas del Toro*] - MCZ**: 1 ♂ 74632 Boquete R.P. 23.03.1941; 1 ♀*Sericopelma* sp. Panama, Chiriquí, Volcán, 1200m el [elevation], 9/viii/1983, H.+L. Levi, in hole in low stone wall. **MUIP**: 1 ♂ *Sericopelma* sp. Finca del las Flores/Fleurs, Boquete Chiriquí Prov. Panama 07 Junio 1968 coll. Jorge Tovan; 1 ♂ *Sericopelma* sp. Boquete, Chiriquí Provence Panama 21.07.1992, coll. Miguel Bogante; 1 ♀ *Sericopelma* sp. Nueva California, Volcan, Chiriqui Province Republic of Panama coll. Leonardo Yanguez, Ríos de Unas Matas de Caña? debajo de las hojas secas suelo humedo; 1 Imm ♂ *Sericopelma* sp. Isla Colon Bocas del Toro Province, Panama 24.10.82 coll Astenid Araiz; 1 ♀ *Sericopelma* sp. Volcán, Province Chiriquí, Republic of Panama, 9.Sept.1987, coll. Arsenio Araug; 1 ♀ *Sericopelma* sp. Isla Taboga, Republic of Panama, Augusto 1985, coll. Kyle Summers. **OUMNH**: 1 ♂ *Sericopelma commune* Panama, Jar 106 Chiriquí, Champion, syntype. [***Panamá, Unknown Prov.*] - MNHN**: 1 ♂ *Sericopelma rubronitens* Ausserer 1875. (Simon det) AR4803 PARIS. **MCZ**: 1 ♂ *Sericopelma* sp.74369, Panama; 2 ♂ *Sericopelma* sp. 74370, Prob[ably] Panama; 1 ♀ 1 Imm ♂*Sericopelma* sp. 74372, Panama.. **MUIP**: 1 ♂ *Sericopelma* sp. Panama 06.12.02 coll. G. Lover; 1 ♂ *Sericopelma* sp. [labeled Theraphosidae] Mulio 9 de Mayo 1994, ?Estuooeucauterisis, Disele el 15 Sept 1993 total 176 dias; 1 ♀ No Data, Panama; 1 Imm ♂ *Sericopelma* sp. San Cristobal ?Verani(S) Sanmig Velito Panama, 10.05.93; 1 ♂ *Sericopelma* sp. Poblado ?Irhe en la Hibrochet, de Banyo 10.00am, Panama, Republic of Panama, 29.12.88 coll. Abraham Beauville.. **OUMNH**: 1 ♀ *Sericopelma* sp. Panama, Jar 82 S. Tinter, Roger, O.P. Cambridge coll. [***Unknown – likely Panama***]: 1 ♂ *Sericopelma* sp. Central America ex London Zoo. [***Costa Rica*] - BMNH**: 1 ♀ *Sericopelma* sp. 1906.11.3.1, Banana River 15 miles from Coast, July 1905, Costa Rica, Jose (?Río Banano, Cantón Limón, Prov. Limón]; 1 ♀ *Sericopelma* sp. 98-12-24-22 Pozo Azul de Pirrís, Prov. San José, Costa Rica, coll. C. Zeledon [Modern Cantón Parrita, Prov. Puntarenas]; **OUMNH**: 1 ♂ *Sericopelma* sp. Guápiles, Pococi, Prov. Limón, Costa Rica. coll. 2006 Viteslav Honsa SERGU1, died n.11.10, ex Benoit Menart; 1 ♀ *Sericopelma* sp. Sabanilla, Puntarenas Sud, Coto Brus, Costa Rica Coll. 2006 leg. Viteslav Honsa SERSB1 died 25.08.10; 1 ♂ as prev. leg. Viteslav Honsa SERSB2 died n.11.10, ex Benoit Menart; 1 ♂ *Sericopelma immensum* 2009 007, pet trade, K Matzen, 1 ♀ *Sericopelma* sp. 2009 007, Costa Rica, ex pet trade w/c 1997; 1 ♂ *Sericopelma melanotarsum* 2009 007, ex pet trade, K Matzen; 1 ♀ *Sericopelma melanotarsum* 2009 007, Costa Rica, w/c died 2000, donated anon. [***Nicaragua*] - MCZ**: 1 ♂ *Sericopelma* sp. 74625 Matagalpa Nicaragua 1073, Richardson Dec 1907, R.C. Feb 12-1909.

***Brachypelma* specimens**:

***Brachypelma albiceps* - BMNH**: 1 ♀ *Brachypelma albiceps* (CB from German import as *Brachypelma ruhnaui*) RUHZ, died VIII.03, Ex E. Hijmensen/ S. Longhorn. **CNAN**: 1 ♂ *Brachypelma albiceps* n79. Teloloapan, Mpio. de Teloloapan. Edo. Guerrero, México. 15.IX.52, coll. Leonila Vazquez; 1 ♂ *Brachypelma albiceps* Presa Vicente Guerrero, Edo. Guerrero, México. 23.XI.96, coll. A. Castido Octavio; 1 ♀ *Brachypelma albiceps* Ref.3094. Presa Vicente Guerrero, Edo. Guerrero, México. 5.X.76. coll. No data. **LAAHFC**: 1 ♂ *Brachypelma albiceps* Sur Morelos. Edo. Morelos 1996. coll. A. Locht. **SJLC** [all pet-trade of unknown providence unless otherwise stated]: 1 Imm *Brachypelma albiceps* RUH2, died 11.IV.09, Ex unknown; 1 ♂ *Brachypelma albiceps* RUH3, died 2008, Ex Mark Davies.

***Brachypelma auratum* - BMNH**: 1 ♀ *Brachypelma auratum* Ref.27. Entre Hermiltepec y Río Pungarancho, Edo. Guerrero, México. 02.XI.2002. coll. E. Gonzalez y C. Duran. **OUMNH**: 4♀ *Brachypelma auratum* 2007 064 pet trade, Lee Arden (spidershop UK) died 2007. **SJLC**: 1 ♀ *Brachypelma auratum* AUR7 died 11.V.08, Ex unknown; 1 ♀ *Brachypelma auratum* AUR9, died VI.03, Ex Paul Herbert; 1 ♂ *Brachypelma auratum* AUR10, died I.04, Ex Stephen Copley; 1 ♂ *Brachypelma auratum* AUR11, died VI.04, Ex Ian Metcalfe; 1 ♂ *Brachypelma auratum* AUR12, died 25.VI.08, Ex Andy Fischer; 1 ♂ 1 ♀ *Brachypelma auratum* AUR13/AUR14, died 2008, Ex Mike Fletcher; 1 ♀ *Brachypelma auratum* AUR15, died 2010, Ex Becky Norris.

***Brachypelma albopilosum* – OUMNH**: 1♀ *Brachypelma albopilosum* 2007 064, pet trade, C/B. **MCZ**: 2 ♀ Brachypelma cf. albopilosum 74614, Georgia Fruit Company, Honduras, 20.v.1932; 1 ♀ Brachypelma cf. albopilosum, 74624, With Fruit Honduras; 1 ♀ Brachypelma cf. albopilosum 74615, Leon River Valley, East of Río Ulúa, Honduras, 1924, Donor, United Fruit Company, 1 ♀ Brachypelma cf. albopilosum Río Ulúa, Tela, Honduras, Fruit Company. **SJLC**: 1 ♂ Brachypelma cf. albopilosum ALB1, died 17.II.01, Ex Ronald Baxter; 1 ♂ Brachypelma cf. albopilosum ALB9, died 03.V.01, Ex Mark Dean; 1 ♂ Brachypelma cf. albopilosum died 01.XII.07, Ex Stuart Longhorn CB; 1 ♂ Brachypelma cf. albopilosum ALB12, died V.08, Ex Andy Matthews. (Plus several further specimens collected across Honduras to be detailed elsewhere).

***Brachypelma baumgarteni* – OUMNH**: 1♀ *Brachypelma baumgarteni* 2007 064 pet trade, Ex Boris Striffler from first import; 1♂ *Brachypelma baumgarteni* 2007 064, pet trade, Ex Boris Striffler from first import - remains of male eaten by female. **SJLC**: 1 imm *Brachypelma baumgarteni* BAU7, died XII.01, Ex Ronald Baxter; 1 ♀ *Brachypelma baumgarteni* BAU8, died I.03, Ex Ronald Baxter; 1 ♀ *Brachypelma baumgarteni* BAU12, died V.08, Ex Paul Herbert; 1 ♂ BAU13, died 2008, Ex Mark Pennell. ***Known hybrid*** - 1 ♂ imm Brachypelma sp. baumgarteni × boehmei, BAU10, died 2008, Ex Eddy Hijmensen.

***Brachypelma boehmei* – SJLC**: 1 ♂ *Brachypelma boehmei* BOH9, died 16.X.03, Ex Ronald Baxter; 1♀ *Brachypelma boehmei* BOH10, died 07.VI.12, Ex Ronald Baxter; 1 ♂ *Brachypelma boehmei* BOH11, died 25.V.08, Ex Mark Kent; 1 ♂ *Brachypelma boehmei* BOH12, died 2001, Ex Mark Dean; 1 imm ♂ *Brachypelma boehmei* BO13, died 2004, Ex Ray Gabriel.

***Brachypelma emilia* – CNAN**: 1 ♀ *Brachypelma emilia* Ref.3121. Sinaloa, Edo. Sinaloa, México. 30.I.65, coll. Ent 46; 1 ♀ *Brachypelma emilia* Ref.3080/No.80. Mazatlán, Mpio. Mazatlán, Edo. Sinaloa, México. VII.1959. coll. Ent 5. **OUMNH**: 2♀ *Brachypelma emilia* 2007 064, pet trade, imported Lee Arden Spidershop UK died 2007; 1♂ 2007 064, *Brachypelma emilia*, pet trade; 1♂ *Brachypelma emilia*, Jar 65, Cambridge coll. (discussed in text). **SJLC**: 1 ♀ *Brachypelma emilia* EME8, died 12.08.09; 1 ♂ *Brachypelma emilia* EME9, died 26.04.09, Ex Andy Fisher; 1 ♀ *Brachypelma emilia* EME10, died 19.05.09, Ex Becky Norris [spermathecae shown in figure 13]; 1 ♂ *Brachypelma emilia* EME11, died 2009, Ex Becky Norris; 1 imm, *Brachypelma emilia* EME12, died 2009, Ex Becky Norris.

***Brachypelma klaasi* – CNAN**: 1 ♂ *Brachypelma klaasi*. Reserva Biosfera Chamela - Cuixmala. Edo. Jalisco, México. 18.V.81. coll. A. Pescador. **LAAHFC**: 1 ♂ *Brachypelma klaasi* Ref.1714.23 Reserva Biosfera Chamela - Cuixmala. Edo. Jalisco, México. 3.IV.98. coll. A. Locht. **OUMNH**: 1♂ *Brachypelma klaasi* 2007 064; 1♂ *Brachypelma klaasi* 2008 071, Eddy Hijmensen. **SJLC**: 1 ♂ *Brachypelma klaas*i KLA1, died IV.01, Ex Mark Pennell; 1 ♂ *Brachypelma klaasi* KLA4, died 08.XII.98, Ex Ray Gabriel; 1 ♂ *Brachypelma klaasi* KLA5, died 2002; 1 ♀ *Brachypelma klaasi* died 2005, Ex Paul Herbert, 1 imm, *Brachypelma klaasi* died 2009, Ex Becky Norris; 1 ♀ *Brachypelma klaasi* died 20.II.10, Ex Mark Pennell.

***Brachypelma schroederi* – SJLC**: 1 ♂ *Brachypelma schroederi* SHR3, died VII.08, Ex Steffan Schroeder; 1 ♂ *Brachypelma schroederi* SHR4, died 01.I.10, Ex Andy Hood; 1 ♂ *Brachypelma schroederi*, SHR6, died 20.IX.15, Ex Peter Roach; 1 ♀ Brachypelma cf. schroederi, SHR5, died 19.V.09, Ex James Box.

**Brachypelma aff. ‘smithi’ – CNAN**: 1 ♀ Brachypelma aff. smithi Entre Tepames y Rio Coahuayana, Frontera, Edo. Colima, México. 8.VII.2005, coll A. Cervantes y M. Olson. **LAAHFC**: 1 ♂ Brachypelma aff. smithi Colima. Edo. Colima, México. 23.Oct. coll. A. Locht; 1 ♂ *Brachypelma smithi* Acapulco, Mpio. Acapulco de Juárez, Edo. Guerrero, México. 20.III.97. coll. A. Locht. **OUMNH**: 1♂ Brachypelma aff. smithi, 2007 064, C/B, 1♂ Brachypelma aff. smithi 2009 001, pet trade, 24/09/08, Yinnon Dolev; **SJLC**: 1 ♀ Brachypelma cf. smithi (traded as *annitha*), ANN1, died 25.III.02, Ex Tony Packer C/B; 1 Imm Brachypelma cf. smithi (traded as *annitha*) ANN2, died 2004, Ex Eddy Hijmensen C/B; 1 ♂ Brachypelma cf. smithi (trade as *annitha*), ANN3, died 2007, Ex Eddy Hijmensen C/B; 1 ♂ Brachypelma aff. smithi SMI1, died IV.01, ex Jean-Michel Verdez; 1 ♂ Brachypelma aff. smithi SMI8, died 03.V.07, Ex Stuart Longhorn; 1 ♂ Brachypelma aff. smithi SMI13, died unk.II.02, Ex Alan Smith; 1 ♂ Brachypelma aff. smithi SMI16, died 2008, Ex Ray Gabriel; 1 ♂ Brachypelma aff. smithi SMI17, died 2008, Ex anon; 1 ♂ Brachypelma aff. smithi SMI18, died 18.V.11, Ex Nicola Dolby; 1 ♀ Brachypelma aff. smithi SMI9, died 31.I.15, Ex Stuart Longhorn; 1 ♀ Brachypelma aff. smithi SMI12, died X.01, Ex Paul Herbert; 1 imm Brachypelma aff. smithi SMI14, died 2004, Ex Ray Gabriel; 1 imm Brachypelma aff. smithi SMI15, died 20.07.07, ex Lee Arden; 1 ♂ Brachypelma aff. smithi × (?) klaasi hybrid, HYBAXB, died II.10, Ex S.Longhorn.

**Brachypelma aff. ‘vagans’ – BMNH**: 1 ♀ *Brachypelma* sp (*vagans*-complex) 2003-148 Las Cuevas Research Station. Chiquibul National Park, Cayo District, Belize. 27.05.01. leg. Stuart Longhorn, Julie Chuter, Martin Nicholas. 1 ♀ *Brachypelma* sp (*vagans*-complex), 2003-148 Pooks Hill Lodge, near Teakettle, Cayo District, Belize, 05.06.01, leg. Stuart Longhorn, Julie Chuter, Martin Nicholas. Plus several other specimens to be detailed elsewhere. **CNAN**: 1 ♂ Brachypelma cf. vagans Celestún, Edo. Yucatán, México. 26.X.00. coll. Tila M.P; 1 ♂ *Brachypelma* sp. (*vagans*-complex) Valerio Trujano, Mpio. Cuicatlan, Edo. Oaxaca, México. 31.XII.04. coll. B. Chavez; 1 ♂ *Brachypelma* sp. (*vagans*-complex) Sierra de Sta. Martha. Los Tuxtlas. Edo. Veracruz, México. 17-XII-76. coll. H. Perez; 1 ♀*Brachypelma* sp. (*vagans*-complex) Área Protegida del Selva Lacandona, Mpio. Ocosingo, México. 7-IV-05. coll. O. Francke, A. Ballesteros, A. Valdez; 2 ♂ *Brachypelma* sp. (*vagans*-complex) Locale as previous. 6.VIII.2005. coll. As previous. **MCZ**: 1 ♀ *Brachypelma* sp. (*vagans*-complex) 74611, Stann Creek, Belize, under rock, 26.vi.1975, W. Sedgewick. 4 Imm *Brachypelma* sp. (*vagans*-complex) 74627, El Cayo, British Honduras [Belize], under rock, Feb.-Mar. 1931; 1 ♀ 3 Imm *Brachypelma* sp. (*vagans*-complex) 74620, Uaxactun, Petén, Guatemala, Mar.-April 1931, H.H. Bartlett. **LAAHFC**: 1 ♀ *Brachypelma* sp. (*vagans*-complex), Los Tuxtlas. Edo. Veracruz, México. No data. coll Unknown; 1 ♂ Brachypelma cf. vagans Merida, Edo. Yucatán, México. XI.1996. coll. H. Lopez; 1 ♂ *Brachypelma* sp. (*vagans*-complex) Playa el Arroyito. Mpio. Santa María de Huatulco, Edo. Oaxaca, México. 28.XII.98. coll. A. Locht. **OUMNH**: 1♀ *Brachypelma* sp. (*vagans*-complex) 2007 064; 1♀ imm *Brachypelma* sp, Jar 61 1905, Cambridge coll; 1♀ Imm *Brachypelma* sp., Cambridge coll, 1♀ *Brachypelma* sp., “Honduras”, Jar 63 Boston* Nr Belize, O. Pickard-Cambridge colln (perhaps New Boston); 1♂ Brachypelma aff. vagans 2007 064, pet trade, C/B; 1♀ Brachypelma aff. vagans 2008 071, pet trade, C/B; 1♂ & 1♀ *Brachypelma vagans*, Jar 69 Guatemala, Cahabón, F. Sarg, Cambridge coll. **PMY**: 2 ♀ *Brachypelma* sp. (*vagans*-group), Tampico, México, Bis;hop Coll. 1940, in life with red abdomen, colour fades before moulting [were misidentified as *Sericopelma rubronitens*]. **SJLC**: 1 ♀ Brachypelma sp. ‘sabulosum’, Aldea El Remate (casa Don David), nr Flores, Petén, Guatemala. Collected 19.V.08, David Ortiz; 1 ♂ Brachypelma sp. ‘sabulosum’, SAB1, died VII.03; 1 ♂ Brachypelma sp. ‘sabulosum’, SAB2, died 01.III.08, Ex Guatemala, Ronald Baxter; 1 ♀ Brachypelma sp. ‘sabulosum’, SAB3, died 12.VI.11, Ex Guatemala, Ronald Baxter; 1 ♀ Brachypelma cf. sabulosum Parque Nacional Yaxha, Petén, Guatemala, 21.V.08. Coll. David Ortiz and Eduard Hijmensen; 1 ♀ Brachypelma sp. ‘vagans’ VAG3, died 25.XI.00; 1 ♂ Brachypelma sp. ‘vagans’, VAG4, died VII.01; 1 ♂ Brachypelma sp. ‘vagans’ VAG7, died 13.VIII.97, Ex Richard Gallon; 1 ♂ Brachypelma sp. ‘vagans’ died pre 2001, Ex Richard Gallon; 1 ♀ *Brachypelma albopilosum* × Brachypelma ‘vagans’ (maternally purebred *albopilosum*) HYB4, died 2005, Ex S. Longhorn; 1 ♂ Brachypelma sp. ‘angustum’ ANG2, died III.01, Ex Tony Davies; 1 ♂ Brachypelma sp. ‘angustum’ ANG3, died VI.01, Ex unknown; 1 ♀ Brachypelma sp. ‘angustum’ ANG4, died 2003, Ex unknown; 1 ♀ Brachypelma sp. ‘angustum’ ANG5, died 2004, Ex Unknown; 1 ♂ Brachypelma sp. ‘angustum’ ANG6, died 10.01, Ex Guy Tansley; 1 ♂ Brachypelma sp. ‘angustum’, ANG7, died 20.X.04, Ex Unknown.

***Brachypelma verdezi* – LAAHFC**: 1 ♀ *Brachypelma verdezi* (labelled *Brachypelma vagans*) Acapulco, Mpio. Acapulco de Juárez, Edo. Guerrero, México. 1990. coll A. Locht,. **SJLC**: 1 ♀ *Brachypelma verdezi* (traded as *pallidum*), PAL2, died 2005; 1 ♀ *Brachypelma verdezi* (traded as pallidum), PAL4, died 26.X.09, Ex David James [see fig. 14]; 1 ♂ *Brachypelma verdezi* PAL5, died 27.IV.09, 1 ♂ *Brachypelma verdezi* PAL6, died 24.09.08, Ex Yinnon Dolev.

***Megaphobema* specimens**:

***Megaphobema mesomelas* - BMNH**: 2 ♂ *Megaphobema mesomelas* (18)96.3.20.3-4, El Azahar, (Canton Cartago, Dept.) Cartago, Costa Rica. J.F. Tristán (Museo Nacional de Costa Rica). 1 ♂ *Megaphobema mesomelas* 1898.12.24.56, La Palma (Prov. Cartago), Costa Rica. J.F. Tristán (Museo Nacional de Costa Rica). [note, this specimen was mislisted as type by Smith 1991b, also has additional label ‘Visto por de Pikelin y Schiapelli, mayo 1968’); 1 ♀ *Megaphobema mesomelas* (18)96.10.25.1 La Palma (Prov. Cartago), Costa Rica. [incl. dissected spermathecae labeled A.M.Smith]; 2 ♂ *Megaphobema mesomelas* 1905.3.29.14-15, Cariblanco (San Carlos, Prov. Alajuela), Costa Rica. Charles H. Lankester [both ex dried/pinned]; 2 ♂ *Megaphobema mesomelas* Dried/pinned collection (possibly additional specimens from H. Rogers, Caché listed by FOPC 1897, Costa rica). **OUMNH**: 1 ♂ *Megaphobema mesomelas* O. Pickard-Cambridge coll, Dried - drawer 46, Costa Rica (possibly syntype examined by FOPC); 2 ♀ *Megaphobema mesomelas* 2007 064, pet trade, Eddy Hijmensen; 1♀ *Megaphobema mesomelas* 2009 001, Costa Rica, Dutch Pet trade; 1 ♂ *Megaphobema mesomelas* 2009 001, Costa Rica, pet trade; 1 ♂ *Megaphobema mesomelas* 2008 072, Costa Rica, 01/12/97, w/c. **SJLC**: 1 ♀ *Megaphobema mesomelas* MES4, died 12.12.01 [see fig. 16].

***Megaphobema peterklaasi* – OUMNH**: 1 ♀ *Megaphobema peterklaasi* 2008 072, pet trade, Ex A Mathews; 1♂ *Megaphobema peterklaasi* 2009 001, Costa Rica, Holland previously dried; 1 ♂ *Megaphobema peterklaasi* 2008 072, Costa Rica, 01/12/97, w/c Costa Rica. **SJLC**: 1 ♀ *Megaphobema peterklaasi* PEK2, died approx 2006, Ex Eddy Hijmensen.

***Megaphobema robustum* – MCZ**: 1 ♂ *Megaphobema robustum* 74298 Colombia, Dept. Meta, Carimagua, 370m, 18 May 1973, Mary Corn, in cow pasture Savannah. **OUMNH**: 4 ♀ *Megaphobema robustum* 2008 072, Colombia, w/c 1997 [see fig. 15 for spermathecae of ROB3, 1 ♀ *Megaphobema robustum* O.P. Cambridge coll, Drawer 9; 1 ♀ *Megaphobema robustum*, Hope / Westwood coll, Drawer 23; 1♀ *Megaphobema robustum*, O.P. Cambridge coll, Drawer 46. **SJLC**: 1 ♂ *Megaphobema robustum*, Colombia, pet trade, died 2013 don. Craig Mackay; 1 ♀ *Megaphobema robustum* ROB5, died n.II.2014, Ex Stuart Longhorn; 1 Juv. *Megaphobema robustum* ROB5, died n.XI.2001, Ex Stuart Longhorn.

***Megaphobema teceae* – OUMNH**: 1 ♂ *Megaphobema teceae* 2008 072, Brazil, Manaus, died 01/01/08 Ex Ken Matzen; 1 ♀*Megaphobema teceae*, 2009 001, Brazil, Manaus, died 01/01/09, Ex K. Matzen 2008.

***Megaphobema velvetosoma* – OUMNH**: 1 ♂ *Megaphobema velvetosoma* VEL7, died 12.06.09, Ex Ray Gabriel; 1 ♀ *Megaphobema velvetosoma* 2008 072, W/c Ecuador (importer Erato Holland) 1997 w/c., 1 ♀ *Megaphobema velvetosoma*, VEL6, died 18.04.12 Ecuador, Ex John Chambers. **SJLC**: 1 ♀ *Megaphobema velvetosoma* VEL5, died 25.IX.15, Ex Stuart Longhorn; 1 imm *Megaphobema velvetosoma* VEL1, died 15.07.01, Ex WC import Paul Stevens, Tena, Ecuador.

***Theraphosa* specimens**:

***Theraphosa apophysis* – BMNH**: 5 ♀& 1 ♂ *Theraphosa apophysis* Venezuela, Roraima. Coll. Ian Wallace. 13.11.90, det. A.M. Smith. **OUMNH**: 3 ♂ *Theraphosa apophysis*, 2007 064, pet trade, c/b.

***Theraphosa stirmi* – BMNH**: 1 ♀ *Theraphosa stirmi* [labelled *Theraphosa blondi*] 1939.3.24.42 British Guiana (=Guyana), New River (East Berbice-Corentyne). Jan-March 1938, Purch. C.A.Hudeson; 1 ♂ & 1 ♀ *Theraphosa stirmi* [labelled *Theraphosa blondi*] Carl Davis. No data; 1 ♂ *Theraphosa stirmi* [labelled *Theraphosa blondi*] British Guiana (=Guyana). 2°19’05.0”N 59°22’33.5”W (=Upper Takutu-Upper Essequibo, Region of Isherton, south Rupununi), G.McDonnell 1933; 1 ♀ *Theraphosa stirmi* [labelled *Theraphosa blondi*] Guyana, Cuyuni-Mazaruni, Upper Waruma. 11.8.1971. M.Lyes Coll. (=British Roraima Expedition), det as *Theraphosa blondi* by W.Bücherl; 1 ♀ *Theraphosa stirmi* [labelled *Theraphosa blondi*] Guyana, (Cuyuni-Mazaruni), Upper Mazaruni, wet trail after rain 3500 ft. British Roraima Expedition 27.8.1971. M.Lyes Coll. det as *Theraphosa blondi* by W.Bücherl; 1 juv. British Roraima Expedition, M.Lyes coll. det. *Theraphosa blondi* by W.Bücherl; 1 ♀ Theraphosa cf. strimi [labelled *Theraphosa blondi*] Brazil, State of Amazonas, Environs of Yanomami village of Watoriki, close to (Rio) Demini, FUNAI post. 1°31’N 62°49’W. **OUMNH**: 1 ♂ Imm *Theraphosa stirmi* (accessioned as sp., Guyana) 2007 064, 10/03/09, Imported Lee Arden (Spidershop UK); 1 ♀*Theraphosa stirmi* [sold as sp. burgundy] 2009 007, Guyana, 10/03/09, Imported Lee Arden (Spidershop UK); 1 ♀ Imm *Theraphosa stirmi* (as *Theraphosa* sp, Guyana) 2009 007, 10/03/09, Imported Lee Arden Spidershop UK; 1 ♀ Imm *Theraphosa stirmi* [sold as sp. burgundy], 2009 051, Guyana, Imported Lee Arden (Spidershop UK).

***Theraphosa blondi* – OUMNH**: 1 ♂ *Theraphosa blondi* 2009 007, pet trade, Guy Tansley; 1 ♀ *Theraphosa blondi* 2009 001, pet trade, c/b 1996 died 01/10/08 K Halsey; 1 ♀ *Theraphosa blondi*, Hope coll; 1 ♂ *Theraphosa blondi* O.P.Cambridge coll, Drawer 48; 1 ♂ Imm *Theraphosa blondi* 2007 064, pet trade, M Walters stuck in moult. **SJLC**: 1 ♀ *Theraphosa blondi*, died 2013. W/c French Guiana, ExJ.M. Verdez.

***Theraphosa* sp. (indet). – BMNH**: ♀ *Theraphosa* sp. indet. [labelled *Theraphosa leblondi*] 1968.2.27.10. British Guiana (=Guyana). Coll. J.W Lester; 1 ♀ *Theraphosa* indet. [labelled as *Theraphosa blondi*] Brazil, St. Paulo (?). S.Roburn. No further data; 1 ♂ *Theraphosa* indet. [labelled *Theraphosa blondi*] No further data.

**Other specimens**:

**PMY**: 1 Imm ♂ *Pamphobeteus*? sp. Guayaquil, Ecuador, Banana Distribution Company, New Haven, 30.iii.1951 [was misidentified by Petrunkevitch as *Sericopelma rubronitens*]; 1 ♀ *Phormictopus* sp, Yale-Fla. Haiti Exped. Feb., March, April 1959 P. S. Humphrey, [was misidentified by Petrunkevitch as *Sericopelma rubronitens*].

**MNHN**: 1 ♀ Theraphosinae indet, AR 4850 MNHN (Simon Collection), ‘Panama and Guatemala’ leg. unknown (same jar as holotype for *Sericopelma panamense*).
